# Influence of hepatitis B virus on the prevalence of diabetes complications in patients with type 2 diabetes

**DOI:** 10.1111/jdi.13954

**Published:** 2022-12-12

**Authors:** Xi‐yu Liu, Yan Zhou

**Affiliations:** ^1^ Department of Endocrinology Dongyang People's Hospital Dongyang China

**Keywords:** Diabetes, Hepatitis B virus, Macrovascular complications

## Abstract

**Aims/Introduction:**

Diabetes and hepatitis B are both global problems. The influence of diabetes on complications and prognosis of hepatitis B has been widely studied. However, the association between hepatitis B virus (HBV) infection and the prevalence of diabetes‐related complications is less documented and is uncertain.

**Materials and Methods:**

This was a retrospective study. We collected information from a large clinical database. A total of 1,090 Chinese inpatients with type 2 diabetes were included.

**Results:**

The participants were divided into two groups, including 135 patients with HBV infection and 955 patients without HBV infection. Patients with HBV infection were younger and had worse control of blood glucose than those without HBV infection. No significant difference was found in the prevalence of diabetic retinopathy, neuropathy, nephropathy, diabetic ketosis or diabetic ketoacidosis between the patients with HBV infection and the patients without HBV infection. The prevalence of macrovascular complications was 54.1% and 64.4% in diabetes patients complicated with HBV infection and without HBV infection, respectively. The *P*‐value was <0.05. However, through the logistic regression analysis, we found HBV infection was not an independent risk factor for macrovascular complications of diabetes.

**Conclusion:**

There was no significant correlation between the prevalence of macrovascular complications, microvascular complications of diabetes, diabetic ketosis or diabetic ketoacidosis and HBV infection status.

## Graphical abstract

There was not a significant difference in the prevalence of diabetic retinopathy, diabetic neuropathy, diabetic nephropathy, diabetic ketosis or diabetic ketoacidosis between the patients with hepatitis B virus and those without hepatitis B virus. There was a significant difference in the prevalence of macrovascular complications between the two groups. Logistic regression analysis showed that hepatitis B virus infection status was not an independent risk factor for macrovascular complications of diabetes patients.

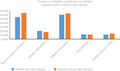

## INTRODUCTION

Globally, the incidence of diabetes is increasing year by year. According to epidemiological findings, it was estimated that 10.5% of adults suffered from diabetes in 2021. Diabetes is a chronic disease, and its complications can lead to death and disability. Approximately 670 million people died of diabetes or its complications, accounting for 12.2% of all global deaths in 2021^1^. Hepatitis B also afflicts people around the word, but the burden of disease is mostly in Asia and Africa^2^, ^3^. According to current research results, the relationship between hepatitis B virus (HBV) infection and the incidence of diabetes is still controversial. Most studies showed that there was no association between the incidence of diabetes and HBV infection^4^, ^5^. In some specific populations, the incidence of diabetes might be related to HBV infection. The studies found that liver cirrhosis was an independent risk factor for diabetes in patients with chronic hepatitis B. The incidence of diabetes was significantly higher in patients with hepatitis B‐related liver cirrhosis than in those without hepatitis B‐related liver cirrhosis^6^, ^7^. It was found that up to 28% of patients with dual infection of HBV and hepatitis C virus had type 2 diabetes^8^. In a cohort study of 439,708 asymptomatic participants undergoing health screening examinations and with a relatively lower prevalence of diabetes (3.8%), participants with HBV infection had 18% higher odds of diabetes compared with those without HBV infection^9^. Among the pregnant women with HBV infection, 6.48% women had diabetes, and among the pregnant women without HBV infection, 3.41% women had diabetes. HBV infection is a risk factor for diabetes during pregnancy^10^.

Diabetes patients with HBV infection were found to have a higher risk of hepatocellular carcinoma and higher mortality^11^, ^12^, ^13^. However, the association between HBV infection and the complications of diabetes is less documented and is uncertain. In the present study, we examined the association between HBV infection and the prevalence of diabetes‐related complications in a cohort of Chinese patients with type 2 diabetes.

## STUDY DESIGN AND POPULATION

This was a single‐center, retrospective and observational study. We analyzed data extracted from a clinical database containing all medical information of patients in our hospital. Our research was in compliance with the Declaration of Helsinki. The study was approved by the Ethics Committee of Dongyang People's Hospital, which waived the requirement for informed patient consent given the retrospective nature of the study. All researchers will keep patient information confidential to maintain the privacy of the participants.

We screened the records of 2,190 patients who were hospitalized in the endocrinology department of our hospital and the primary diagnosis was diabetes. Among these, we excluded the following individuals: 389 cases of acute infectious diseases; 157 cases of uremia; 50 patients with other kidney diseases, except diabetic nephropathy; 80 patients with other type of diabetes, except type 2 diabetes; 11 pregnant patients; and 412 patients without comprehensive evaluation of diabetic complications or detection of HBV. Ultimately, there were 1,090 cases for statistical analysis.

### Inclusion criteria and exclusion criteria

The inclusion criteria were: (i) diagnosis of type 2 diabetes according to the World Health Organization standard; (ii) hospitalized patients with evaluation of diabetes‐related complications; and (iii) detection of HBV.

The exclusion criteria were: (i) acute infectious diseases; (ii) uremia; (iii) other kidney diseases, except diabetic nephropathy; (iv) other type of diabetes, except type 2 diabetes; and (v) pregnancy and lactation.

### Detection method and evaluation of diabetes complications

Glycosylated hemoglobin was measured using high‐performance liquid chromatography^14^ (Bio‐Rad Laboratories, Marnes la Coquette, France). Blood glucose, uric acid, triglyceride, total cholesterol, liver function and kidney function were measured using colorimetric/fluorometricassay^15^, ^16^ (Fuji Wako, Osaka, Japan). C‐reactive protein levels were.

measured using the immunoturbidimetric method. The glomerular filtration rates were estimated by the Modification of Diet in Renal Disease formula^17^. Insulin and C‐peptide levels were measured using the electrochemiluminescence method (Roche Diagnostics, Shanghai, China). Hepatitis B surface antigen was tested using commercially available enzyme‐linked immunosorbent assays (Abbott Diagnostika, Wiesbaden‐Delkenheim, Germany).

The evaluation of diabetes complications referred to the American guideline^18^, ^19^. Macrovascular complications of type 2 diabetes was defined as coronary heart disease, stroke or peripheral arterial diseases, including carotid arteriosclerosis, lower limb arteriosclerosis, the history of myocardial infarction and stroke in the present study. Diabetic retinopathy was evaluated by an ophthalmologist or optometrist with the discharge diagnosis of diabetic retinopathy. Diabetic neuropathy was diagnosed by nerve conduction velocity test, regardless of symptoms. Diabetes nephropathy was evaluated by creatinine and urine microalbumin.

### Statistical analysis

IBM SPSS Statistics 25.0 (IBM Corp., Armonk, NY, USA) was used for statistical analysis. The independent samples *t*‐test was used for comparison of continuous variables. The Kruskal–Wallis test was carried out before the independent samples *t*‐test between the groups. The rates between two groups were compared by the χ^2^‐test. Logistic regression analysis was used to identify the association between variables.

## RESULTS

In this cohort of 1,090 Chinese inpatients with type 2 diabetes, the mean age was 58.6 ± 13.4 years, and 725 (66.5%) patients were men. The proportion of diabetes patients complicated with HBV infection was 12.4%. Table 1 shows the clinical characteristics and metabolic profiles of the participants. The patients with HBV infection were younger and had worse control of blood glucose than those without HBV infection. The patients with HBV infection had higher total bilirubin levels and a higher proportion drank alcohol than those without HBV infection.

**Table 1:** Basic demographic and clinical characteristics of participants

Variables	Patients with HBV infection (*n* = 135)	Patients without HBV infection (*n* = 955)	*P*
Age (years)	56.2 ± 11.9	59.0 ± 13.6	0.022*
Duration of diabetes (years)	5.4 ± 3.7	6.2 ± 4.2	0.029*
Status of smoking (%)			
Smoking cessation	2.2%	2.7%	0.662
Smoking	39.3%	36.2%	
No smoking	58.5%	61.1%	
Status of drinking (%)			
Drinking cessation	0.7%	0.9%	0.014*
Drinking	45.2%	34.3%	
No drinking	54.1%	64.7%	
BMI (kg/m^2^)	24.4 ± 3.9	24.6 ± 3.4	0.545
HbA1c (%)	9.8 ± 2.8	9.2 ± 2.6	0.012*
Systolic blood pressure (mmHg)	132 ± 22	134 ± 23	0.515
Diastolic blood pressure (mmHg)	81 ± 12	81 ± 13	0.898
Uric acid (μmol/L)	310 ± 109	323 ± 95	0.170
Total cholesterol (mmol/L)	4.28 ± 0.93	4.35 ± 1.21	0.456
Triglycerides (mmol/L)	1.92 ± 1.63	2.01 ± 2.10	0.654
ALT (U/L)	46 ± 118	29 ± 28	0.088
TBIL (μmol/L)	13.5 ± 8.0	11.8 ± 5.7	0.019*
Creatinine (μmol/L)	64.7 ± 34.7	70.1 ± 36.6	0.108
Fasting C peptide (ng/mL)	1.89 ± 1.24	1.95 ± 1.09	0.538

* *P*‐value is <0.05.

ALT, alanine aminotransferase; BMI, body mass index; HbA1c, glycosylated hemoglobin; HBV, hepatitis B virus; TBIL, total bilirubin.

The association of HBV infection with various indicators was assessed by logistic regression analysis, as shown in Table 2. We found that there was no association between the status of HBV infection and metabolic indicators in diabetes patients including, glycosylated hemoglobin, blood pressure, body mass index, total cholesterol, total triglycerides and urinary microalbumin.

**Table 2:** Correlation analysis of hepatitis B virus infection with metabolic indicators in diabetes patients

Variables	*B* value	SE value	*P*‐value
Age (years)	−0.011	0.009	0.195
BMI (kg/m^2^)	−0.021	0.032	0.515
HbA1c	0.071	0.043	0.096
Systolic blood pressure (mmHg)	0.000	0.005	0.983
Uric acid (μmol/L)	0.000	0.001	0.950
Total cholesterol (mmol/L)	−0.113	0.103	0.273
Triglycerides (mmol/L)	0.015	0.056	0.792
Creatinine (μmol/L)	−0.003	0.004	0.433
TBIL(μmol/L)	0.031	0.015	0.041*
ALT (U/L)	0.003	0.002	0.112

* *P*‐value is <0.05.

ALT, alanine aminotransferase; BMI, body mass index; HbA1c, glycosylated hemoglobin; HBV, hepatitis B virus; TBIL, total bilirubin.

**Figure 1:**
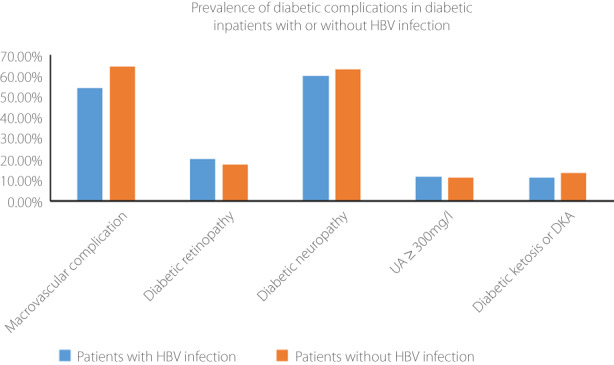
A total of 1,090 Chinese inpatients with type 2 diabetes were included in this study. The participants were divided into two groups, including 135 patients with hepatitis B virus (HBV) infection and 955 patients without HBV infection. There was a significant difference in the prevalence of macrovascular complications between the two groups. *P*‐value <0.05 was considered statistically significant.

We investigated the prevalence of diabetes complications in diabetes patients with or without HBV infection. The participants were divided into two groups, the diabetes patients with HBV infection and the diabetes patients without HBV infection. The results are shown in Table 3 and Figure 1. No significant difference was found in the prevalence of diabetic retinopathy, neuropathy, diabetic ketosis or diabetic ketoacidosis between the two groups. Urinary microalbumin and serum creatinine are two key factors used to assess the presence of diabetic nephropathy. These showed that no significant difference was found in the incidence of diabetic nephropathy between the diabetes patients with HBV infection and the diabetes patients without HBV infection. However, diabetes patients with HBV infection statistically had a lower prevalence of macrovascular complications than the those without HBV infection. The *P*‐value was <0.05.

**Table 3:** Prevalence of diabetic complications in patients with or without hepatitis B virus infection

Diabetic complications	Diabetic patients with HBV infection (*n* = 135)	Diabetic patients without HBV infection (*n* = 955)	*P*
Macrovascular complication	73 (54.1%)	615 (64.4%)	0.020*
Diabetic retinopathy	27 (20.0%)	167 (17.4%)	0.475
Diabetic neuropathy	81 (60.0%)	604 (63.2%)	0.465
Normal urinary microalbumin	86 (63.7%	634 (66.4%)	0.567
UA 30–300 mg/L	33 (24.4%)	215 (22.5%)	
UA ≥300 mg/L	16 (11.9)	106 (11.1%)	
Serum creatinine (μmol/L)	64.7 ± 34.7	70.1 ± 36.6	0.108
Diabetic ketosis or DKA	15 (11.1%)	127 (13.3%)	0.480

* *P*‐value is <0.05.

DKA, diabetic ketoacidosis; HBV, HBV, hepatitis B virus; UA, urinary microalbumin.

Logistic regression analysis was used to identify the association of macrovascular complications with variables of diabetes patients. Table 4 shows that the macrovascular complications were associated with age, sex, creatinine and total bilirubin. HBV infection was not an independent risk factor for macrovascular complications of diabetes patients.

**Table 4:** Correlation analysis of macrovascular complication in diabetes inpatients

Variables	OR	95% confidence interval	*P*‐value
Age (years)	1.117	1.096–1.138	0.000*
Duration of diabetes (years)	1.010	0.967–1.054	0.662
BMI (kg/m^2^)	0.977	0.929–1.028	0.369
HbA1c	1.006	0.933–1.084	0.876
Systolic blood pressure (mmHg)	1.007	0.999–1.016	0.073
Uric acid (μmol/L)	1.001	0.999–1.004	0.235
Total cholesterol (mmol/L)	0.912	0.776–1.071	0.261
Triglycerides (mmol/L)	1.041	0.950–1.141	0.391
Creatinine (μmol/L)	0.992	0.986–0.998	0.010*
UA grading	1.039	0.803–1.344	0.773
TBIL (μmol/L)	0.960	0.933–0.989	0.007*
ALT (U/L)	1.002	0.998–1.006	0.244
Sex (male)	2.059	1.264–3.353	0.004*
HBV infection status	0.755	0.464–1.228	0.258
Smoking status	1.183	0.802–1.745	0.396
Drinking status	1.145	0.786–1.669	0.479

*R* ^2^ (coefficient of determination, Cox & Snell), 0.262; *R* ^2^ (Nagelkerke), 0.358.

* *P*‐value is <0.01.

ALT, alanine aminotransferase; BMI, body mass index; HbA1c, glycosylated hemoglobin; TBIL, total bilirubin; UA, urinary microalbumin.

## DISCUSSION

Diabetes patients complicated with HBV infection were found to have a higher risk of complications of hepatitis B. However, the association between HBV infection and the prevalence of diabetes‐related complications is less documented and is uncertain. Diabetes‐related complications include macrovascular complications, microvascular complications and acute complications, such as diabetic ketosis and diabetic ketoacidosis. We found that diabetes patients with HBV infection had a lower prevalence of macrovascular complications than those without HBV infection. However, the logistic regression analysis results showed that HBV infection was not an independent risk factor for macrovascular complications among diabetes patients. In the present study, patients with HBV infection were younger than those patients without HBV infection. Age is an independent risk factor for macrovascular complications of type 2 diabetes. Older diabetes patients had a higher prevalence of cardiovascular diseases than the younger diabetes patients^20^. This could explain why the regression analysis results (shown in Table 4) were inconsistent with the *t*‐test results (in Table 3) in the present study. One study found that chronic HBV infection was associated with increased cardiovascular risks^21^. This seems to be inconsistent with our findings. Their study population consisted of 97 type 2 diabetes patients with chronic renal insufficiency (the median serum creatinine was up to 200 μmol/L). In the present study, the mean serum creatinine level was 64.7 μmol/L in the group with HBV infection and 70.1 μmol/L in the group without HBV infection. Patients with kidney disease had worse cardiovascular outcomes^22^. The different conclusions might be due to the different study populations.

We found that there was not a significant difference in the prevalence of microvascular complications between the diabetes patients with HBV infection and those patients without HBV infection. Gisi *et al*.^23^ also found that there was no relationship between HBV infection and the microvascular complications of diabetes. Their results was consistent with the present findings. However, a prospective study showed that HBV infection was an independent risk factor for renal prognosis among type 2 diabetes patients^24^. The incidence of end‐stage renal diseases (defined as requiring dialysis, with serum creatinine exceeding twofold the upper limit of normal, or serum creatinine ≥500 μmol/L) was compared between the participants with HBV infection and the participants without HBV infection in the aforementioned research. The present study excluded end‐stage renal diseases. This could explain why we came to different conclusions.

In conclusion, diabetes patients complicated with HBV infection had a lower prevalence of macrovascular complications than those without HBV infection in the present study cohort. Through regression analysis, we found HBV infection was not an independent risk factor for macrovascular complications in diabetes patients. No significant difference was found in the incidence of microvascular complications, diabetic ketosis or diabetic ketoacidosis between the diabetes patients with HBV infection and those without HBV infection.

### CLINICAL PERSPECTIVES

For patients with diabetes mellitus complicated with hepatitis B, the present study can guide the decision‐making of diabetes complications evaluation. Our study showed that there was no significant correlation between the prevalence of macrovascular complications, microvascular complications, diabetic ketosis, diabetic ketoacidosis and HBV infection. For patients with diabetes mellitus complicated with hepatitis B, the frequency of evaluating diabetes‐related complications can be the same as those without HBV infection.

## DISCLOSURE

The authors declare no conflict of interest.

Approval of the research protocol: This study conformed to the provisions of the Declaration of Helsinki. It was approved by the Ethics Committee of Dongyang People's Hospital.

Informed consent: Informed consent was waived given the retrospective nature of the study.

Registry and the registration no. of the study/trial: N/A.

Animal studies: N/A.

## Data Availability

Data are openly available in a public repository that issues datasets with DOIs. The data that support the findings of this study are openly available in Figshare. DOI:10.6084/m9.figshare.20296563.
